# An open-source tool to identify active travel from hip-worn accelerometer, GPS and GIS data

**DOI:** 10.1186/s12966-018-0724-y

**Published:** 2018-09-21

**Authors:** Duncan S. Procter, Angie S. Page, Ashley R. Cooper, Claire M. Nightingale, Bina Ram, Alicja R. Rudnicka, Peter H. Whincup, Christelle Clary, Daniel Lewis, Steven Cummins, Anne Ellaway, Billie Giles-Corti, Derek G. Cook, Christopher G. Owen

**Affiliations:** 1 Centre for Exercise, Nutrition and Health Sciences, University of Bristol, 8 Priory Road, Bristol, BS8 1TZ UK; 2 National Institute for Health Research Bristol Biomedical Research Centre, University Hospitals Bristol NHS Foundation Trust and University of Bristol, Bristol, UK; 3 Population Health Research Institute, St George’s, University of London, London, UK; 4 Department of Social and Environmental Health Research, London School of Hygiene and Tropical Medicine, London, UK; 5 MRC/CSO Social & Public Health Sciences Unit, University of Glasgow, Glasgow, UK; 6 NHMRC Centre for Research Excellence in Healthy Liveable Communities, Centre for Urban Research, RMIT University, Melbourne, Australia

**Keywords:** Machine learning, Xgboost, Active travel, Travel mode, Physical activity, GPS, Accelerometer, Gradient boosting

## Abstract

**Background:**

Increases in physical activity through active travel have the potential to have large beneficial effects on populations, through both better health outcomes and reduced motorized traffic. However accurately identifying travel mode in large datasets is problematic. Here we provide an open source tool to quantify time spent stationary and in four travel modes(walking, cycling, train, motorised vehicle) from accelerometer measured physical activity data, combined with GPS and GIS data.

**Methods:**

The Examining Neighbourhood Activities in Built Living Environments in London study evaluates the effect of the built environment on health behaviours, including physical activity. Participants wore accelerometers and GPS receivers on the hip for 7 days. We time-matched accelerometer and GPS, and then extracted data from the commutes of 326 adult participants, using stated commute times and modes, which were manually checked to confirm stated travel mode. This yielded examples of five travel modes: walking, cycling, motorised vehicle, train and stationary. We used this example data to train a gradient boosted tree, a form of supervised machine learning algorithm, on each data point (131,537 points), rather than on journeys. Accuracy during training was assessed using five-fold cross-validation. We also manually identified the travel behaviour of both 21 participants from ENABLE London (402,749 points), and 10 participants from a separate study (STAMP-2, 210,936 points), who were not included in the training data. We compared our predictions against this manual identification to further test accuracy and test generalisability.

**Results:**

Applying the algorithm, we correctly identified travel mode 97.3% of the time in cross-validation (mean sensitivity 96.3%, mean active travel sensitivity 94.6%). We showed 96.0% agreement between manual identification and prediction of 21 individuals’ travel modes (mean sensitivity 92.3%, mean active travel sensitivity 84.9%) and 96.5% agreement between the STAMP-2 study and predictions (mean sensitivity 85.5%, mean active travel sensitivity 78.9%).

**Conclusion:**

We present a generalizable tool that identifies time spent stationary and time spent walking with very high precision, time spent in trains or vehicles with good precision, and time spent cycling with moderate precisionIn studies where both accelerometer and GPS data are available this tool complements analyses of physical activity, showing whether differences in PA may be explained by differences in travel mode. All code necessary to replicate, fit and predict to other datasets is provided to facilitate use by other researchers.

**Electronic supplementary material:**

The online version of this article (10.1186/s12966-018-0724-y) contains supplementary material, which is available to authorized users.

## Background

Non-communicable diseases, such as cardiovascular disease, type 2 diabetes and cancer, account for almost half of the adult disease burden worldwide [1]. The importance of physical activity in decreasing the burden of such chronic diseases is well-established [2], and increasing the physical activity of populations has become a key goal of public health policy [1, 3, 4]. Active travel, predominantly by walking and cycling, is an accessible form of physical activity, which is associated with positive health outcomes [5–7]. Quantifying the proportion of time spent in different active travel modes is therefore important to understand how these contribute to overall physical activity and health, and to assess the effectiveness of interventions that aim to increase active travel. Assessment of active travel changes, alongside quantification of PA levels, will aid understanding in both observation and intervention based studies.

Travel modes have previously been assessed using detailed travel diaries [8]. However, self-reported data have limitations, because they may be subject to social desirability and recall bias and often record only a single day of travel [9]. Activity and movement patterns are now increasingly objectively assessed, using accelerometers and GPS receivers [10–12]. Combining accelerometer and GPS data allows for the identification of both the intensity and location of physical activity. This combination of activity and location is potentially valuable to describe travel behaviour, and particularly active travel behaviour.

Accelerometry has been widely used, with many different devices deployed. The most common devices in the literature are the ActiGraph, Actiheart, Actical, activePAL and GeneActiv, of which over half of published studies used the ActiGraph, up to 2015 [13]. Many of these different devices convert raw acceleration (measured in g) into some form of activity count variable, which has been used to classify physical activity intensity and energy expenditure [14–17]. However, the methods used to convert raw acceleration into counts are often unclear. For this reason, and because the raw acceleration data contain much more information to train an algorithm than derived count variables, we have made use of the raw data in this study.

Previous work on travel mode identification has been developed from the transport perspective rather than physical activity, where segmentation into journeys is important to assess travel behaviour [18–21]. A focus on journeys often results in short periods of physically active transit behaviour, such as walking between bus-stops, being identified as part of a non-active travel mode. However, for physical activity researchers, quantifying the volume and intensity of physical activity when actively travelling is an essential component of the overall purpose of a journey. Consequently, it is important to identify all data-points that denote active travel, so that all time in active travel modes can be quantified. As a result, we identify the travel mode of each GPS data point (recorded every 10 s), without prior segmentation into journeys.

In recent years, supervised machine learning has shown the potential to identify active travel from physical activity data. Supervised machine learning algorithms are trained on an example data-set, and are then used for prediction to other data-sets. The most promising algorithms appear to be random forests, an ensemble supervised learning algorithm where predictions are taken from a consensus across a large number of decision trees [18, 22, 23]. A related algorithm, gradient boosted trees, has recently replaced random forests as a leading algorithm for data science tasks, with many machine learning approaches using the XGBoost implementation of gradient boosting instead of random forests [24, 25].

Here we present a method to distinguish five travel modes (walk, cycle, motorised vehicle, train, and stationary) using accelerometer and GPS data and the supervised machine learning tool XGBoost. We use survey data from the Examining Neighbourhood Activities in Built Living Environments in London (ENABLE London) study [26] to create a training data-set of combined GPS and accelerometer data of daily commuters on which to develop and test the algorithm. All code necessary to replicate our findings and apply our predictive model to other data-sets is made available as a package of the open-source statistical software environment R [27, 28]. We also provide a full usage example, so that researchers inexperienced in coded input tools such as R can apply the model [28].

## Methods

### Data collection

We used data from the ENABLE London study, which is described in detail elsewhere [26]. In brief, the study is examining the effect of the area of residence, including features of the local built and social environment on health behaviours, particularly physical activity levels. Between January 2013 and December 2015, a total of 1278 adult participants were recruited from neighbourhoods largely in the east of London, UK.Participants were asked to wear an accelerometer (ActiGraph GT3X+; Florida, USA) and a GPS receiver (Qstarz BT-1000XT; Taipei, Taiwan) on an elasticated belt worn around their waist for seven consecutive days, removing devices for sleep, swimming and bathing, with 1089 (85%) participants providing both accelerometer and GPS data. Participants also completed a questionnaire to describe their travel patterns to work/place of study. They reported the specific days on which they would be travelling to work/study during the ActiGraph and GPS wear period, and whether they commuted at the same time on each day. Reported travel modes for these journeys to and from work were: tube (underground) / train (overground) / bus, minibus or coach / taxi / motorcycle, scooter or moped / driving a car or van / passenger in a car or van / bicycle / walk / jog / other. Travel modes were re-categorised into walk, cycle, vehicle (taxi, motorcycle, car/van driver and car/van passenger/bus/minibus/coach) and train (underground and overground rail) for this analysis (Table 1). Insufficient participants consistently jogged to work for us to be able to separate “jog” as an additional mode. In addition, time of leaving and arriving for each journey to and from work was collected. The study was approved by the City Road and Hampstead Ethical Review Board (REC reference number 12LO1031); all participants gave written informed consent.

**Table 1:** The characteristics of the training data and how we classify travel modes

Commute Mode	Number of participants	% of total	Training category
Walk	66	20.2	Walk
Cycle	34	10.4	Cycle
Car/Van driver	48	14.7	Vehicle
Car/Van passenger	6	1.8	Vehicle
Motorcycle/ moped/ scooter	1	0.3	Vehicle
Taxi	2	0.6	Vehicle
Bus/minibus/coach	37	11.3	Vehicle
Train (over ground)	46	14.1	Train
Underground	86	26.4	Train
Total	326	100	–

### Data preparation and cleaning

Raw accelerometer data were extracted as csv files using ActiLife 6 software (ActiGraph, Florida, USA). We chose to not use processed accelerometer count data, because count data is processed differently for each device, and so is not comparable between devices. Furthermore, raw accelerometer data is more detailed than count data, allowing us to create more variables for our algorithm to assess. Acceleration data were then summarised per 10 s epoch as the median absolute deviation from the median, 10th percentile, 90th percentile, skewness and kurtosis of each axis of the accelerometer. We calculated the fast Fourier transform of the accelerometer signal and took the mean strength of all acceleration signals for each accelerometer axis per 10 s epoch. Mean strength was extracted following visual inspection of the full range of transforms, with all showing a similar pattern between travel modes. These derived accelerometer characteristics were merged by timestamp to GPS data using a custom R function [28]. We identified non-wear time using the GGIR 1.5–12 package of R, which identifies periods of 60 min where there is a standard deviation on at least two accelerometer axes of less than 13 mg (1 mg  =  0.00981 ms^− 2^) [29, 30]. A wide variety of non-wear time algorithms have been used, and a 60 min window is recommended to balance accuracy of non-wear time identification with minimising data loss [31]. The 60-min window we use is analogous to that used on count data, but uses small axis deviations instead of count data, because our analysis focusses on raw data. To provide a measure of satellite signal quality we calculated the sum of the signal to noise ratio (sumSNR) from each satellite the GPS device was connected to at each epoch. SumSNR is a measure of the accuracy of signal coming from each satellite to the GPS device – if the signal is unobstructed then there should be many satellites connected to the GPS device, and each should show a high signal to noise ratio. If there are obstructions to the GPS signal, such as the participant being indoors, then there will be fewer satellites connected to the GPS device, with lower signal to noise ratios. SumSNR gives a single measure of signal quality, rather than the three measures (horizontal, vertical and position dilution of precision) the GPS device outputs, therefore reducing the number of variables to consider.

During normal travel, variables such as speed or accelerometer signals are not stable across every 10 s epoch, there will be variation, e.g., cars must stop for traffic lights, walkers must pause to cross roads, GPS signal can be poor inside trains, leading to loss of accuracy where single data-points can be located over 50 m from train lines. This variation makes all modes more difficult to identify. We smoothed out some of the inherent natural variation in travel by calculating four-minute moving windows of the calculated accelerometer variables. For each window we calculated mean, standard deviation, 10th and 90th percentile of each accelerometer axis and speed, from the GPS device. We also calculated mean sumSNR and mean distance from train lines. We calculated the distance of each data-point from train lines using a combination of Meridian 2 rail network data for the UK, OS OpenMAP data for central London and the *spatstat* package of R [32–34]. If researchers are not used to using GIS data, it is currently more easy to get hold of and utilise than ever before, and included within our code is a demonstration of the acquisition and use of train line data [28]. We also calculated distance travelled over the previous minute and over the next minute. All the variables were chosen because they are likely to differ between travel modes: vehicles and trains should have higher speed than walking and cycling; walking should show greater accelerometer activity than other modes; cycling may show higher accelerometer activity than vehicles and trains; both vehicles and trains have metal structures around the participant which may obstruct GPS signal resulting in lower sumSNR than other travel modes. Either distance over the next minute or distance over the previous minute should be very low while a subject is stationary.

### Training data-set creation

The purpose of the training data is to provide reliable examples of how different travel modes are represented in accelerometer and GPS data, at 10 s epochs. As a result, it is important that a data-point in the training data is a true representation of the assigned travel mode. The context around the training data is less relevant, i.e. the purpose is to ensure that a data point is taken from a point in time when someone is walking; whether they were recently in other types of travel behaviours is not relevant to the assignment to a specific travel mode. As a result, we use a conservative methodology to extract reliable points from commute journeys. Importantly, this does not mean that our method can only predict the mode of commutes, rather we train the model using data from commutes and predict to all data.

The time of the journey to and from work was extracted from the ENABLE London questionnaire, using the participants reported home departure time and work arrival time (commute to work) and work departure time and home arrival time (commute from work). We only used participants who commuted to and from work using the same mode of transport and who specified the time they usually commuted for both journeys, in total 326 participants (Table 1, Additional file 1 for demographics). We extracted all combined GPS and accelerometry data during commutes in R and exported this as a shapefile for ArcGIS 10.4, using the *sp* and *rgdal* packages of R [35, 36]. Assuming a five-day working week for each participant, the total possible number of commutes included within our training data is therefore 3260 (two commutes per day for 5 days). However, not all data were available due to non-wear, GPS signal loss and participants not necessarily working 5 day weeks: hence, our training data-set was based on 1174 commutes from the 326 participants.

**Fig. 1:**
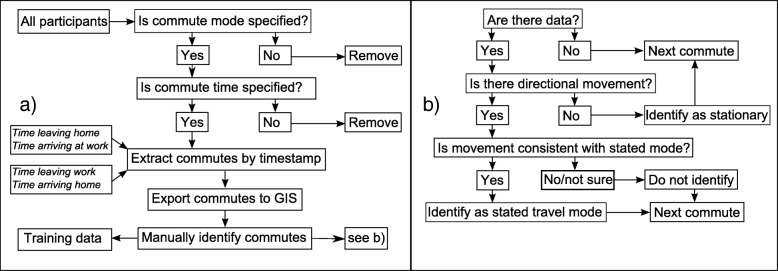
**a** Workflow for creation of training data-set, **b**) decisions made to manually identify commutes

We then manually identified all points during (total 131,537) the commute of each participant that conformed to the mode they stated, using a Geographical Information System (ArcGIS 10.4) (Fig. 1a). Only the points relevant to the stated mode were identified, i.e., if the participant used a train and walked to and from the train station we only marked those points along the train line as the mode “train”, and the walked points would not be included in the training data (Fig. 1b). Any sequences of points where we could not clearly ascertain what travel mode was in use, e.g. because there was repeated GPS signal loss, were excluded from the training data. In addition, any points occurring within the period of the commute that showed no directionality and were clustered around a single location were classified as “stationary”. As a result, each participant contributed to the mode which they stated they used to commute and, potentially, to the “stationary” mode, if they displayed any non-travel behaviour. Vehicle and train journeys were confirmed by location of appropriate features (e.g., roads/rail tracks). The identification of the “stationary” subset allowed us to predict travel mode for every data point in the full data-set, without first making assumptions about which points represent any form of travel. It is important to note that stationary does not imply inactive. For our purposes we are seeking to identify when participants travel, therefore walking within a building, which will appear stationary in terms of GPS signal, would be classified as stationary using our methodology. This twofold assessment of the training data, both with the participants stating their mode during this time, and then a researcher manually double checking it in GIS, means that we can be sure that our training data-set contains reliable examples of each travel mode.

### Model fitting and prediction

The training data-set was split into two different sections for model-fitting. First, we extracted a subset of the training data to test different moving window sizes: if a participant contributed multiple days to the training data, we took the first day to test moving windows. If a participant only contributed a single day to the training data, we extracted half of that day’s data to test moving window sizes. Secondly, we used the remaining points to fit a gradient boosted tree, using the XGBoost package of R [37]. Gradient boosted trees are an example of an ensemble learner [38]. To learn how to assign a mode to each point, a gradient boosted tree fits a large number of decision trees to the data, each of which is shallow. Following creation of a decision tree, data-points are re-weighted (the relative importance of points are changed), to emphasize points which were miss-classified last round, and a new decision tree is fit. Each tree is a weak learner i.e. it performs better than random but with poor accuracy, however a consensus across all trees leads to high predictive accuracy. In prediction, each point is assessed for how likely they are to belong to each travel mode, with the highest probability determining the predicted outcome.

Where our parameters differ from default, it is to avoid overfitting to the training data as much as possible. Therefore the learning rate is set lower than default (0.1 instead of 0.3). The learning weight is a measure of how much we dilute the default re-weighting of points. We also used subsampling value of 0.2, so that each tree was only fit to 20% of the available data.

We used five-fold cross-validation to assess model accuracy [39]. Participants within the training data were randomly assigned to one of five subsets. Each subset was iteratively removed from the pool of training data, a model was trained on the remaining 80% of the training data and the excluded subset was used as test data. Therefore, our cross-validation contains five separate fitted models and test data-sets, of which we report the overall accuracy scores. Full cross-validation output is available in Additional file 2, along with all model parameters used.

While our training data contains reliable examples of different travel modes when participants are known to be travelling, it is not a true representation of identifying travel from free-living data. As a further test of the predictive accuracy of our method, we compared our predicted mode with the manually identified travel patterns across all time periods from other participants. We randomly selected 21 participants from the ENABLE London study, who contributed 402,749 data points. These participants were not included within the training data-set. We exported each individual’s data as a Shapefile for ArcGIS 10.4 and then worked through each day of data, manually classifying the travel-mode of every data-point.

To test the generalisability of our fitted model to other data-sets we also compared predicted values to manually identified data from 10 participants from a second separate data-set (total 210,936 data points), the Sedentary Time and Metabolic Health in People with type-2 Diabetes study (STAMP-2) [40]. Briefly, STAMP-2 was a cross-sectional observational study of sedentary behaviour in adults with newly diagnosed (diagnosis within previous 5–12 months) type-2 diabetes, conducted in two English National Health Service (NHS) Foundation Trusts in South West England. A total of 139 participants were recruited between January 2014 and June through diabetes education days, general practitioner (GP) referral and self-referral. Eligibility criteria were: aged 30 to 70 years, a clinical diagnosis of T2DM (HbA1c > 48 mmol/mol; > 6.5%) within the previous five to 12 months, no ketosis and a body mass index (BMI) of > 25 kg/m^2^. Exclusion criteria were unstable angina, a myocardial infarction within the previous 3 months and a medical condition that precludes PA (e.g. a foot ulcer). Participants wore an ActiGraph accelerometer for 7 days, and those in one centre also wore a GPS receiver for the same period. STAMP-2 received ethical approval from the South West-Central Bristol NHS Research Ethics Committee (13/SW/0187). All participants provided written informed consent before taking part in the study.

STAMP-2 participants were independent of the ENABLE London study and were recruited from a city with different travel options from central London. Furthermore, the STAMP-2 participants had been recently diagnosed with type-2 diabetes and represented an older (age mean, sd = 58.6, 8.6), less healthy demographic (70.1% obese, BMI mean, sd = 34.4, 7.3), than those in the ENABLE London study. Good predictive performance on this data-set would demonstrate that the algorithm can generalise to other populations and geographical contexts.

### Measures of test performance

We report several measures of predictive accuracy in this paper. Firstly, we present an overall accuracy score: the percentage of points we correctly predict overall. This is tempting because it reduces the overall prediction accuracy down to a single number. However, if you have uneven numbers of data-points in each category, which we do, this is not necessarily representative of each mode we report. We therefore also report positive predictive value (the percentage of those points we predict as a mode that were observed as that mode), sensitivity (the percentage of points observed to be a travel mode, which we correctly predict) and F1 score (the harmonic mean of positive predictive value and sensitivity) for each mode separately. To fully understand how our model performs all of these values are useful. For those unfamiliar with such terms we recommend interpreting the F1 score for each mode as a measure of accuracy for that mode. For researchers who would like more information we also present the raw confusion matrix. This table compares counts for observed and predicted mode, and can also be used to calculate accuracy scores.

## Results

### Training data

We identified 66 participants from the ENABLE study who walked to work, 34 cyclists, 94 vehicle users (grouping together car/van drivers, car/van passengers, taxi users, bus/coach users and motorcyclists) and 132 train users (grouping together underground and over ground train users) (Table 1). In total, the training data-set contained 131,573 data-points (365.5 h): 12,791 walking (35.5 h), 11,607 cycling (32.2 h), 29,407 in vehicles (81.7 h), 18,269 train (50.7 h) and 59,499 ‘stationary’ (165.3 h). These training data were split into two parts, as described above. The subset of the data used to test moving window sizes contained approximately a quarter of the training data (33,529 points). Following testing of 1, 2, 3, 4 and 5-min moving windows, we selected a four-minute window, because a four-minute window resulted in the highest predictive accuracy for active travel modes (Additional file 3). The remaining 98,387 points were used to build the cross-validated model.

### Model prediction

In model cross-validation, overall, we correctly predicted 97.3% of points. All five travel modes were predicted with high accuracy (lowest F1 score 93.9; Table 2). The F1 score is the harmonic mean of positive predictive value and sensitivity, therefore a high value represents a high rate of correct identification of both true positives and true negatives.

**Table 2:** The confusion-matrix and accuracy scores per mode, expressed as percentages, for the cross-validated model

		Observed mode					Mode	Positive predictive value^a^	Sensitivity^b^	F1 score^c^
		Cycle	Walk	Train	Vehicle	Stationary				
Predicted mode	Cycle	8062	33	5	328	20	Cycle	95.4	96.2	95.8
	Walk	10	9171	21	32	357	Walk	95.6	92.3	93.9
	Train	3	18	12,296	95	18	Train	98.9	97.5	98.2
	Vehicle	260	6	139	20,911	159	Vehicle	97.4	96.9	97.1
	Stationary	47	710	155	214	45,317	Stationary	97.6	98.8	98.2

^a^Positive predictive value (PPV) or Precision, the ratio of true positives to the sum of true and false positives

^b^Sensitivity or Recall/ True positive rate/Detection rate, the ratio of true positive to true positives and false negatives

^c^F1 score, the harmonic mean of PPV and sensitivity

In comparison with manually identified data (*n* = 21), overall accuracy was still high at 96.0% of predictions being correct, however, this was substantially driven by the fact that most people spent most of their time stationary (83.7% of the time stationary). Our F1 scores for the other modes were lower than in cross-validation, with the lowest at 75.5 for cycling (Table 3).

**Table 3:** The confusion-matrix and accuracy scores per mode, expressed as percentages, compared with manually-identified data

		Observed mode					Mode	Positive predictive value^a^	Sensitivity^b^	F1 score^c^
		Cycle	Walk	Train	Vehicle	Stationary				
Predicted mode	Cycle	3651	1426	116	215	385	Cycle	63.0	94.3	75.5
	Walk	15	23,346	12	128	1858	Walk	92.1	75.6	83.0
	Train	43	749	6275	89	1318	Train	74.1	96.6	83.8
	Vehicle	156	585	38	23,684	3726	Vehicle	84.0	97.1	90.1
	Stationary	8	4776	58	285	329,807	Stationary	98.5	97.8	98.2

^a^Positive predictive value (PPV) or Precision, the ratio of true positives to the sum of true and false positives

^b^Sensitivity or Recall/ True positive rate/Detection rate, the ratio of true positive to true positives and false negatives

^c^F1 score, the harmonic mean of PPV and sensitivity

When compared with manual identification of travel mode from the STAMP-2 study, our predictions performed well considering the different participants and context (Table 4). We correctly predicted the travel mode of 96.5% of points, but again this is driven by our high accuracy on stationary points, which is the dominant mode (86.8% of time stationary). The poorest performing predictive mode was for cycling, with an F1 score of 69.1% (Table 4).

**Table 4:** The confusion-matrix and accuracy scores per mode, expressed as percentages, compared with the STAMP-2 study

		Observed mode					Mode	Positive predictive value^a^	Sensitivity^b^	F1 score^c^
		Cycle	Walk	Train	Vehicle	Stationary				
Predicted mode	Cycle	1381	290	0	109	182	Cycle	70.4	67.9	69.1
	Walk	17	7729	0	1	202	Walk	97.2	73.6	83.8
	Train	1	39	716	0	320	Train	66.5	89.8	76.5
	Vehicle	602	329	0	14,242	2840	Vehicle	79.1	98.1	87.5
	Stationary	33	2112	81	171	179,539	Stationary	98.7	98.1	98.4

^a^Positive predictive value (PPV) or Precision, the ratio of true positives to the sum of true and false positives

^b^Sensitivity or Recall/ True positive rate/Detection rate, the ratio of true positive to true positives and false negatives

^c^F1 score, the harmonic mean of PPV and sensitivity

To understand, how the model miss-classifies in some situations we also present the total time predicted and observed in each travel mode (Table 5). In cross-validation the times are remarkably close, however in the other comparisons there are notable differences. Total time walking is underestimated based on predictions, and vehicle time is over-estimated.

**Table 5:** Time observed and predicted per participant in each travel mode for the different data-sets

Travel mode	Mean minutes in travel mode per participant					
	Cross validation		ENABLE full days		STAMP	
	Reported	Predicted	Manually identified	Predicted	Manually identified	Predicted
Cycle	5.4	5.4	30.7	46.0	33.9	32.7
Walk	6.4	6.1	245.1	201.3	175.0	132.5
Train	8.1	8.1	51.6	67.3	13.3	17.9
Vehicle	13.9	13.8	193.7	223.7	242.1	300.2
Stationary	29.5	29.9	2675.4	2658.2	3051.4	3032.3

## Discussion

We have developed an accurate predictive algorithm, which identifies five travel modes, including the active travel modes walking and cycling, identifying each travel mode correctly over 90% of the time. Our levels of accuracy in cross-validation out-perform recent similar studies [18, 23]. The models developed here are made freely available to apply to similar data in the statistical software environment R [28]. It is unsurprising that our accuracy scores are lower when compared with manual identification of both participants within this study and from elsewhere, than in cross-validation of the training data-set. Identification is most likely to be accurate during defined trips, which is what the training data are comprised of. During full days of data there are likely to be other more ambiguous forms of movement, which may be short in duration, and therefore difficult to identify, or which do not fully represent one of the forms of travel we have included. Our model performs least well for the detection of cycling, and therefore is likely overfitted to our training data. We would therefore recommend manual checking of cycling data, in order to improve accuracy. In this study, cycling represents a relatively small amount of total time, therefore manual checking of this subset represents a much smaller time investment than the full analysis.

Applying the algorithm to the STAMP-2 dataset resulted in similar levels of accuracy of travel mode prediction within a very different group of participants, living in a different environmental setting. This finding suggests that our method may be generalizable to other data-sets and could be used by other researchers without the time-consuming steps of creating new training data. However, until more robust tests have been completed, we would recommend a manual identification checking stage similar to our methods, to verify the generalisability of the method.

We exhibit similar levels of accuracy to the PALMS system, which is a freely available method to process physical activity data [21]. The purpose of the output is somewhat different, though, with PALMS identifying journeys and our method identifying each data point. The preferred method will depend on what the research question is. One advantage our R package does have over the server-based system in PALMS, is that it can be run on a researcher’s own machine. Data used for the present (and other similar) analyses is subject to strict data privacy and ethical conditions. Running the analyses on a researchers’ own machine, rather than uploading to a server for remote processing, can help avoid problems related to data privacy. Furthermore, our method is open source, meaning all code is freely available online [28]. As a result, other researchers can suggest edits and improvement and contribute to the development of our method, and its utility to the research community.

Visual inspection of the data in GIS revealed that much of the disagreement between prediction and manual classification was found at the start and end of journeys. This highlights the challenge of identifying modal shift, i.e. when to switch from one mode to another. This is a limitation of the current method. However, the strength of identifying individual data points, rather than journeys, means that imprecision in identifying modal shift results in small numbers of mis-classified points rather than entire mis-classified journeys. Small numbers of mis-classified points will only have a small effect on total time in each travel mode. Our inaccuracy at the start and end of trips mean that any prediction to a data-set where many short-duration trips are expected in high volume would be expected to yield lower accuracy. Conversely, longer journeys should be able to be identified with greater precision.

It is worth highlighting that some disagreement within our test data-sets may not be true errors. For example, if a participant is stationary, but on a train, it is questionable whether they should be classified as using a train or stationary and it may not matter as long as the rule is applied consistently. However, when comparing the predicted vs manually derived methods this causes disagreement, because during manual classification, stationary points on train lines were termed stationary (for example standing at a train station), yet the algorithm identifies them as “train”. A number of the miss-classifications between our manual identification and predicted data-sets may therefore be open to interpretation, and may not be true miss-classifications..

The prediction of active travel modes should complement existing analyses of physical activity using accelerometers. Accelerometers have been used with great effect to objectively quantify activity, but they are not without limitations. A well-known problem of traditional PA analyses using accelerometers is that cycling is not recognised as a form of moderate to vigorous activity, because cycling generates relatively low readings on a waist-worn accelerometer compared to other active modes. Identification of cycling from combined accelerometer and GPS data will allow better quantification of cycling as a form of physical activity. Traditional PA analyses will still miss cycling as a form of activity, but our method quantifies it, although with moderate precision. Furthermore, assessment of active travel using our method will help understand how people are active. For example, activity at a single location, e.g. the home/gym, will not be classed as active travel using our method, rather as a stationary travel mode, therefore a participant may show high levels of overall physical activity but low levels of active travel. Incorporation of this extra information will help to understand participant’s overall physical activity patterns. For example: if a participant shows increased PA but not active travel it is likely that they have increased their activity at locations such as at home or the gym. If we see no change in PA but an increase in active travel then participants have replaced some of their PA at a location with active travel.

There are several limitations to our study. First, we only identify the active modes walking and cycling, there is no consideration of running, or any other activity. This limitation is based on our study sample, where no commuters consistently used these modes, so we could not include them in our training data. However, walking and cycling represent the most frequently used active transport modes, and other modes were rarely reported by our participants. Any form of running will most likely be identified as walking using our method, due to the high acceleration that running causes on an accelerometer, and so will still be identified as active travel. We therefore feel that this limitation will have little impact on our study, although we would recommend caution in applying our method to a data-set where large quantities of running are expected. Secondly, we have demonstrated our capacity to identify major travel modes, but do not discriminate car travel from public transport. Consequently, if a study is attempting to quantify the use of public transport the current method is inadequate. To address this, we have developed a second model that discriminates bus travel. However, this leads to reduced overall accuracy, because the pattern of speed/activity can be confused between buses and other vehicles (Additional file 4). Further work in this area would need to assess the generalisability of our methods. Thirdly, our method does not identify the purpose of the journey, therefore there is no distinction between leisure and transport-related travel. Another method in addition to our identification step will be necessary to determine the context of the journey. Lastly, though we assess different geographical contexts, both are in the UK. We do not know how well the method would perform elsewhere in the world, where other transport options may be available. Furthermore, we have only tested the current model on adults, and further research could assess how well our model performs on older adults or children, to potentially be of use in a wider group of studies.

## Conclusion

In summary we have developed a method to identify travel modes from accelerometer, GPS and GIS data for the ENABLE London study, which successfully predicts over 90% of points tested in a range of contexts. This method can be of use to complement existing analyses of physical activity, and assess active travel alongside physical activity. All code necessary to replicate the analysis, apply the method to other data-sets or predict from our models to other data-sets are provided, to facilitate usage by other researchers.

## Additional files

Additional file 1: Characteristics of the training data and remainder of the cohort from ENABLE, for further detail and demographics see the baseline cohort paper (Ram et al., 2016, BMJ Open 6: e012643) (DOCX 15 kb)

Additional file 2: Model accuracy for each cross-validation subset (DOCX 26 kb)

Additional file 3: Testing of moving window sizes for predictive accuracy. Model parameters: ETA = 0.1 (a measure of how conservative XGBoost is, set lower than standard to be more conservative and ovoid over-fitting), rounds = 200, subsample = 0.2 (use 0.2 of data in each model, again to avoid over-fitting), max tree depth = 10, gamma = 10, all others default (DOCX 26 kb)

Additional file 4: Fitted model accuracy when buses are included (DOCX 14 kb)
