# Supplementary material for: An open-source tool to identify active travel from hip-worn accelerometer, GPS and GIS data
Source: Int J Behav Nutr Phys Act. 2018 Sep 21;15:91. doi: 10.1186/s12966-018-0724-y (PMC6150970; doi:10.1186/s12966-018-0724-y)
Supplement: Supplementary file 1 — Characteristics of the training data and remainder of the cohort from ENABLE, for further detail and demographics see the baseline cohort paper (Ram et al., 2016, BMJ Open 6: e012643) (DOCX 15 kb) [file 12966_2018_724_MOESM1_ESM.docx]

**Additional file 1.** Characteristics of the training data and remainder of the cohort from ENABLE, for further detail and demographics see the baseline cohort paper (Ram et al., 2016, BMJ Open 6: e012643)

|  | | Number of participants (%) | | Age (years) | |
| --- | --- | --- | --- | --- | --- |
|  |  | Non-training data | Training data | Non-training data | Training data |
| All participants | | 763 (-) | 326 (-) | 33 (10.1) | 33.8 (9.8) |
| Sex | Female | 443 (58.1) | 177 (54.3) | 34 (10.6) | 34.0 (10.5) |
|  | Male | 320 (41.9) | 149 (45.7) | 31.7 (9.2) | 33.5 (8.8) |
| Housing sector | Intermediate | 328 (43) | 159 (48.8) | 31.6 (7.2) | 31.9 (8.6) |
|  | Market Rent | 129 (16.9) | 71 (21.8) | 31.6 (10.9) | 30.0 (8.2) |
|  | Social | 306 (40.1) | 96 (29.4) | 35.3 (11.8) | 39.9 (10.1) |
| Ethnicity | Asian/British Asian | 124 (16.3) | 54 (16.6) | 32.8 (8.1) | 35.4 (9.5) |
|  | Black/ African/ Caribbean/ Black British | 192 (25.2) | 71 (21.8) | 34.5 (11.9) | 38.4 (11.0) |
|  |  |  |  |  |  |
|  | Mixed/Multiple | 36 (4.7) | 20 (6.1) | 30.9 (10.7) | 31.2 (9.4) |
|  | Other | 30 (3.9) | 12 (3.7) | 39.2 (12.7) | 37.2 (5.3) |
|  | White | 381 (49.9) | 169 (51.8) | 32.1 (9.1) | 31.5 (8.8) |
