# Supplementary material for: An open-source tool to identify active travel from hip-worn accelerometer, GPS and GIS data
Source: Int J Behav Nutr Phys Act. 2018 Sep 21;15:91. doi: 10.1186/s12966-018-0724-y (PMC6150970; doi:10.1186/s12966-018-0724-y)
Supplement: Supplementary file 2 — Model accuracy for each cross-validation subset (DOCX 26 kb) [file 12966_2018_724_MOESM2_ESM.docx]

**Additional file 2.** Model accuracy for each cross-validation subset. Model settings identical to Appendix 1

Cross-validation subset 1

|  | | Observed mode | | | | |  | Modes | Positive predictive value | Sensitivity | F1 score |
| --- | --- | --- | --- | --- | --- | --- | --- | --- | --- | --- | --- |
|  |  | Cycle | Stationary | Train | Vehicle | Walk |  |  |  |  |  |
| Predicted mode | Cycle | 1327 | 1 | 0 | 20 | 0 |  | Cycle | 98.4 | 94.7 | 96.5 |
|  | Stationary | 12 | 8295 | 29 | 20 | 410 |  | Stationary | 94.6 | 99.5 | 97.0 |
|  | Train | 0 | 10 | 1636 | 9 | 0 |  | Train | 98.9 | 97.1 | 98.0 |
|  | Vehicle | 62 | 9 | 13 | 5865 | 0 |  | Vehicle | 98.6 | 99.2 | 98.9 |
|  | Walk | 0 | 25 | 6 | 1 | 1713 |  | Walk | 98.2 | 80.7 | 88.6 |

Cross-validation subset 2

|  | | Observed mode | | | | |  | Modes | Positive predictive value | Sensitivity | F1 score |
| --- | --- | --- | --- | --- | --- | --- | --- | --- | --- | --- | --- |
|  |  | Cycle | Stationary | Train | Vehicle | Walk |  |  |  |  |  |
| Predicted mode | Cycle | 2422 | 1 | 3 | 151 | 16 |  | Cycle | 93.4 | 95.9 | 94.6 |
|  | Stationary | 6 | 10268 | 4 | 11 | 107 |  | Stationary | 98.8 | 99.3 | 99.0 |
|  | Train | 0 | 0 | 3072 | 13 | 0 |  | Train | 99.6 | 99.7 | 99.6 |
|  | Vehicle | 90 | 17 | 1 | 1587 | 0 |  | Vehicle | 93.6 | 90.0 | 91.8 |
|  | Walk | 7 | 59 | 2 | 1 | 2411 |  | Walk | 97.2 | 95.1 | 96.2 |

Cross-validation subset 3

|  | | Observed mode | | | | |  | Modes | Positive predictive value | Sensitivity | F1 score |
| --- | --- | --- | --- | --- | --- | --- | --- | --- | --- | --- | --- |
|  |  | Cycle | Stationary | Train | Vehicle | Walk |  |  |  |  |  |
| Predicted mode | Cycle | 1958 | 2 | 0 | 34 | 0 |  | Cycle | 98.2 | 95.9 | 97.1 |
|  | Stationary | 1 | 9552 | 57 | 8 | 29 |  | Stationary | 99.0 | 97.6 | 98.3 |
|  | Train | 0 | 8 | 2314 | 6 | 10 |  | Train | 99.0 | 95.4 | 97.1 |
|  | Vehicle | 79 | 103 | 55 | 4404 | 3 |  | Vehicle | 94.8 | 98.9 | 96.8 |
|  | Walk | 3 | 119 | 0 | 0 | 2026 |  | Walk | 94.3 | 98.0 | 96.1 |

Cross-validation subset 4

|  | | Observed mode | | | | |  | Modes | Positive predictive value | Sensitivity | F1 score |
| --- | --- | --- | --- | --- | --- | --- | --- | --- | --- | --- | --- |
|  |  | Cycle | Stationary | Train | Vehicle | Walk |  |  |  |  |  |
| Predicted mode | Cycle | 393 | 8 | 1 | 103 | 10 |  | Cycle | 76.3 | 95.9 | 85.0 |
|  | Stationary | 0 | 9714 | 37 | 89 | 121 |  | Stationary | 97.5 | 98.3 | 97.9 |
|  | Train | 0 | 0 | 1589 | 44 | 8 |  | Train | 96.8 | 95.7 | 96.3 |
|  | Vehicle | 17 | 14 | 22 | 4855 | 0 |  | Vehicle | 98.9 | 94.8 | 96.8 |
|  | Walk | 0 | 150 | 11 | 30 | 1803 |  | Walk | 90.4 | 92.8 | 91.6 |

Cross-validation subset 5

|  | | Observed mode | | | | |  | Modes | Positive predictive value | Sensitivity | F1 score |
| --- | --- | --- | --- | --- | --- | --- | --- | --- | --- | --- | --- |
|  |  | Cycle | Stationary | Train | Vehicle | Walk |  |  |  |  |  |
| Predicted mode | Cycle | 1962 | 8 | 1 | 20 | 7 |  | Cycle | 98.2 | 97.9 | 98.0 |
|  | Stationary | 28 | 6930 | 10 | 86 | 39 |  | Stationary | 97.7 | 99.6 | 98.7 |
|  | Train | 3 | 0 | 3683 | 23 | 0 |  | Train | 99.3 | 98.4 | 98.8 |
|  | Vehicle | 12 | 14 | 48 | 4200 | 3 |  | Vehicle | 98.2 | 97.0 | 97.6 |
|  | Walk | 0 | 4 | 2 | 0 | 1102 |  | Walk | 99.5 | 95.7 | 97.6 |
