# Supplementary material for: An open-source tool to identify active travel from hip-worn accelerometer, GPS and GIS data
Source: Int J Behav Nutr Phys Act. 2018 Sep 21;15:91. doi: 10.1186/s12966-018-0724-y (PMC6150970; doi:10.1186/s12966-018-0724-y)
Supplement: Supplementary file 3 — Testing of moving window sizes for predictive accuracy. Model parameters: ETA = 0.1 (a measure of how conservative XGBoost is, set lower than standard to be more conservative and ovoid over-fitting), rounds = 200, subsample = 0.2 (use 0.2 of data in each model, again to avoid over-fitting), max tree depth = 10, gamma = 10, all others default (DOCX 26 kb) [file 12966_2018_724_MOESM3_ESM.docx]

**Additional file 3**. Testing of moving window sizes for predictive accuracy. Model parameters: ETA = 0.1 (a measure of how conservative XGBoost is, set lower than standard to be more conservative and ovoid over-fitting), rounds = 200, subsample = 0.2 (use 0.2 of data in each model, again to avoid over-fitting), max tree depth = 10, gamma = 10, all others default

| 1 minute | | Observed mode | | | | |  |  | Positive Predictive Value | Sensitivity | F1 score |
| --- | --- | --- | --- | --- | --- | --- | --- | --- | --- | --- | --- |
|  |  | Cycle | Stationary | Train | Vehicle | Walk |  |  |  |  |  |
| Predicted mode | Cycle | 2708 | 5 | 28 | 221 | 1 |  | Cycle | 91.4 | 84.0 | 87.5 |
|  | Stationary | 88 | 14108 | 75 | 214 | 75 |  | Stationary | 96.9 | 98.1 | 97.5 |
|  | Train | 16 | 129 | 5300 | 218 | 9 |  | Train | 93.4 | 93.8 | 93.6 |
|  | Vehicle | 384 | 107 | 233 | 6506 | 9 |  | Vehicle | 89.9 | 90.6 | 90.2 |
|  | Walk | 29 | 33 | 17 | 24 | 2992 |  | Walk | 96.7 | 97.0 | 96.8 |

| 2 minutes | | Observed mode | | | | |  |  | Positive Predictive Value | Sensitivity | F1 score |
| --- | --- | --- | --- | --- | --- | --- | --- | --- | --- | --- | --- |
|  |  | Cycle | Stationary | Train | Vehicle | Walk |  |  |  |  |  |
| Predicted mode | Cycle | 2765 | 11 | 16 | 246 | 0 |  | Cycle | 91.0 | 85.7 | 88.3 |
|  | Stationary | 78 | 14204 | 59 | 172 | 68 |  | Stationary | 97.4 | 98.8 | 98.1 |
|  | Train | 11 | 2 | 5341 | 167 | 4 |  | Train | 96.7 | 94.5 | 95.6 |
|  | Vehicle | 346 | 112 | 229 | 6582 | 9 |  | Vehicle | 90.4 | 91.6 | 91.0 |
|  | Walk | 25 | 53 | 8 | 16 | 3005 |  | Walk | 96.7 | 97.4 | 97.0 |

| 3 minutes | | Observed mode | | | | |  |  | Positive Predictive Value | Sensitivity | F1 score |
| --- | --- | --- | --- | --- | --- | --- | --- | --- | --- | --- | --- |
|  |  | Cycle | Stationary | Train | Vehicle | Walk |  |  |  |  |  |
| Predicted mode | Cycle | 67 | 14196 | 29 | 121 | 52 |  | Cycle | 91.0 | 86.3 | 88.6 |
|  | Stationary | 4 | 0 | 5371 | 128 | 5 |  | Stationary | 98.1 | 98.7 | 98.4 |
|  | Train | 356 | 122 | 236 | 6683 | 4 |  | Train | 97.5 | 95.0 | 96.2 |
|  | Vehicle | 14 | 50 | 4 | 3 | 3023 |  | Vehicle | 90.3 | 93.0 | 91.6 |
|  | Walk | 0 | 0 | 0 | 0 | 0 |  | Walk | 97.7 | 98.0 | 97.8 |

| 4 minutes | | Observed mode | | | | |  |  | Positive Predictive Value | Sensitivity | F1 score |
| --- | --- | --- | --- | --- | --- | --- | --- | --- | --- | --- | --- |
|  |  | Cycle | Stationary | Train | Vehicle | Walk |  |  |  |  |  |
| Predicted mode | Cycle | 2789 | 23 | 10 | 216 | 10 |  | Cycle | 91.5 | 86.5 | 88.9 |
|  | Stationary | 65 | 14197 | 14 | 102 | 50 |  | Stationary | 98.4 | 98.7 | 98.6 |
|  | Train | 0 | 0 | 5388 | 121 | 4 |  | Train | 97.7 | 95.3 | 96.5 |
|  | Vehicle | 360 | 128 | 237 | 6744 | 0 |  | Vehicle | 90.3 | 93.9 | 92.1 |
|  | Walk | 11 | 34 | 4 | 0 | 3022 |  | Walk | 98.4 | 97.9 | 98.2 |

| 5 minutes | | Observed mode | | | | |  |  | Positive Predictive Value | Sensitivity | F1 score |
| --- | --- | --- | --- | --- | --- | --- | --- | --- | --- | --- | --- |
|  |  | Cycle | Stationary | Train | Vehicle | Walk |  |  |  |  |  |
| Predicted mode | Cycle | 2777 | 27 | 12 | 206 | 15 |  | Cycle | 91.4 | 86.1 | 88.7 |
|  | Stationary | 64 | 13598 | 8 | 105 | 45 |  | Stationary | 98.4 | 99.2 | 98.8 |
|  | Train | 0 | 0 | 5394 | 166 | 5 |  | Train | 96.9 | 95.4 | 96.2 |
|  | Vehicle | 375 | 64 | 236 | 6704 | 0 |  | Vehicle | 90.9 | 93.3 | 92.1 |
|  | Walk | 9 | 23 | 2 | 1 | 2904 |  | Walk | 98.8 | 97.8 | 98.3 |
