# Supplementary material for: An open-source tool to identify active travel from hip-worn accelerometer, GPS and GIS data
Source: Int J Behav Nutr Phys Act. 2018 Sep 21;15:91. doi: 10.1186/s12966-018-0724-y (PMC6150970; doi:10.1186/s12966-018-0724-y)
Supplement: Supplementary file 4 — Fitted model accuracy when buses are included (DOCX 14 kb) [file 12966_2018_724_MOESM4_ESM.docx]

**Additional file 4.** Fitted model accuracy when buses are included

|  | | Observed mode | | | | | |  | Mode | Positive predictive value | Sensitivity | F1 score |
| --- | --- | --- | --- | --- | --- | --- | --- | --- | --- | --- | --- | --- |
|  |  | Cycle | Walk | Train | Vehicle | Bus | Stationary |  |  |  |  |  |
| Predicted mode | Cycle | 8124 | 32 | 3 | 67 | 322 | 17 |  | Cycle | 94.9 | 96.9 | 95.9 |
|  | Walk | 6 | 9186 | 16 | 6 | 28 | 357 |  | Walk | 95.7 | 92.4 | 94.0 |
|  | Train | 3 | 19 | 12322 | 73 | 36 | 25 |  | Train | 98.7 | 97.7 | 98.2 |
|  | Vehicle | 6 | 0 | 76 | 9801 | 1892 | 36 |  | Vehicle | 83.0 | 82.5 | 82.7 |
|  | Bus | 198 | 10 | 66 | 1910 | 7193 | 130 |  | Bus | 75.7 | 74.2 | 74.9 |
|  | Stationary | 45 | 691 | 133 | 30 | 222 | 45306 |  | Stationary | 97.6 | 98.8 | 98.2 |
